# Methodological Concerns in TNFAIP3, IRAK1, and TLR4 Polymorphism Analysis for RA Susceptibility

**DOI:** 10.1002/iid3.70426

**Published:** 2026-05-21

**Authors:** Aditya K. Panda

**Affiliations:** ^1^ ImmGen EvSys Lab, BT‐113, Department of Biotechnology Berhampur University Berhampur Odisha India; ^2^ Centre of Excellence on Bioprospecting of Ethno‐Pharmaceuticals of Southern Odisha (CoE‐BESO) Berhampur University Berhampur Odisha India

Dear Editor,

I read with interest the article by Tang and colleagues, “Analysis of TNFAIP3, IRAK1, and TLR4 Gene Polymorphisms in Patients with Rheumatoid Arthritis,” which examines six SNPs in a Han Chinese population with RA [1]. This topic is important and clinically relevant; however, I find that several aspects of the study design and analyses detract, at least to my mind, from the strength of the conclusions drawn.

First, no sample size or power calculation was reported in the published article. Although multiple SNPs, genetic models, and stratified analyses were performed, no adjustment for multiple testing was made. Various genetic comparison models yielded *p* values of 0.012, 0.020, 0.024, and 0.043, which were treated as definitive, although they may not be, with an adjustment based on basic Bonferroni or false discovery rate calculations. As no multiple corrections were applied to the results, caution is warranted in interpretation [2].

Second, the logistic regression analysis was not clearly described. Cases and controls were matched for age and sex, yet the odds ratios in Table 3 seem largely unadjusted, and it is not stated whether age, sex, and RF/ACPA positivity were accounted for as covariates. This is especially pertinent to IRAK1 rs1059703, as it is an X‐linked SNP in a female‐biased disease. Sex‐appropriate modeling would have made the shown “protective” effect of the A allele more convincing.

Third, the handling of population structures seems limited. Although all individuals are said to be Han Chinese from a single clinical center, there has been no investigation of the possibility of within‐group regional or subethnic variations in prevalence or genetic composition, nor has there been any assessment of relative population structure stratification. In addition, some genotypes appear to be very rare (rs6920220 AA, rs5029930 CC, and rs5029939 GG), leading to weaker Hardy–Weinberg testing and unstable odds ratios. Without replication in an independent population, the findings are limited in generalizability.

Considering these aspects of the study, the conclusion that TNFAIP3, IRAK1, and TLR4 polymorphisms “are associated with RA susceptibility” is perhaps more definitive than the data truly warrant. Acknowledging this investigation as a hypothesis‐generating one, with the intention to replicate in larger, independent populations, may be a more constructive message.

## Author Contributions

**Aditya K. Panda:** conceptualization, supervision, writing – original draft, writing – review and editing, investigation.

## Funding

The author has nothing to report.

## Ethics Statement

The author has nothing to report.

## Conflicts of Interest

The author declares no conflicts of interest.

## References

[1] Z. Tang , L. Peng , Z. Zhao , et al., “Analysis of TNFAIP3, IRAK1, and TLR4 Gene Polymorphisms in Patients With Rheumatoid Arthritis,” Immunity, Inflammation and Disease 14, no. 2 (2026): e70344, 10.1002/iid3.70344. 41684094 PMC12902183

[2] A. K. Panda and B. Mahapatra , “Strengthening Methodological Rigor and Reporting Precision in Genetic Association Studies: A Critique,” Lupus (2026): 1–3, 10.1177/09612033261419943. 41566794

